# Diseased-induced multifaceted variations in community assembly and functions of plant-associated microbiomes

**DOI:** 10.3389/fmicb.2023.1141585

**Published:** 2023-03-16

**Authors:** Lu Kuang, Ting Li, Baozhan Wang, Junwei Peng, Jiangang Li, Pengfa Li, Jiandong Jiang

**Affiliations:** ^1^Key Lab of Microbiology for Agricultural Environment, Ministry of Agriculture, College of Life Sciences, Nanjing Agricultural University, Nanjing, China; ^2^State Key Laboratory of Soil and Sustainable Agriculture, Institute of Soil Science, Chinese Academy of Sciences, Nanjing, China

**Keywords:** plant microbiome, bacterial wilt disease, assembly mechanism, microbial interactions, microbiome function

## Abstract

Plant-associated microorganisms are believed to be part of the so-called extended plant phenotypes, affecting plant growth and health. Understanding how plant-associated microorganisms respond to pathogen invasion is crucial to controlling plant diseases through microbiome manipulation. In this study, healthy and diseased (bacterial wilt disease, BWD) tomato (*Solanum lycopersicum* L.) plants were harvested, and variations in the rhizosphere and root endosphere microbial communities were subsequently investigated using amplicon and shotgun metagenome sequencing. BWD led to a significant increase in rhizosphere bacterial diversity in the rhizosphere but reduced bacterial diversity in the root endosphere. The ecological null model indicated that BWD enhanced the bacterial deterministic processes in both the rhizosphere and root endosphere. Network analysis showed that microbial co-occurrence complexity was increased in BWD-infected plants. Moreover, higher universal ecological dynamics of microbial communities were observed in the diseased rhizosphere. Metagenomic analysis revealed the enrichment of more functional gene pathways in the infected rhizosphere. More importantly, when tomato plants were infected with BWD, some plant-harmful pathways such as quorum sensing were significantly enriched, while some plant-beneficial pathways such as streptomycin biosynthesis were depleted. These findings broaden the understanding of plant–microbiome interactions and provide new clues to the underlying mechanism behind the interaction between the plant microbiome and BWD.

## Introduction

Plant-associated microorganisms play a major role in affecting plant growth and health (Muller et al., 2016; Theis et al., 2016; Singh et al., 2020; Woodhams et al., 2020). The rhizosphere, a hot spot for plants to exchange substances and energy with the surrounding environment, has drawn the most attention (Li et al., 2020; Li P. et al., 2022). There is an emerging consensus about the dominant role of the rhizosphere microbiome in influencing host performance, especially regarding resistance to disease (Berendsen et al., 2012; Li X. et al., 2019); for example, disrupting the balance between the abundances of *Firmicutes* and *Actinobacteria* in the tomato rhizosphere causes increased incidence of bacterial wilt disease (BWD; Lee et al., 2021). Understanding how the rhizosphere microbiome responds to disease incidence is fundamental to understanding the microbiome pathways that improve plant health, potentially allowing the favorable manipulation of the microbiome.

Multiple biotic and abiotic factors contribute to plant microbiome assemblies, such as host genotype, plant growth stage, local climates, regional microbial species pool, soil type, and field management strategies (Berendsen et al., 2012; Gao et al., 2020). Apart from these factors, changes in plant performance caused by a pathogen invasion are considered to be some of the most important forces driving microbiome assembly (Carrion et al., 2019). Species composition and community diversity can be greatly influenced by pathogen invasion (Gao et al., 2021); more plant-beneficial microorganisms, especially those that can antagonize phytopathogens, are enriched in diseased plant organs (Gao et al., 2021), and the alpha diversity of the rhizosphere microbiome significantly declined after pathogen invasion (Li et al., 2021). Our knowledge of whether pathogen invasion also affects other aspects of the plant microbiome, such as microbial interactions, community assembly, and metabolic functions, is limited.

In metacommunity ecology, niche and neutral mechanisms are two divergent, but complementary, paradigms that describe the assemblages of the metacommunity (Zhou and Ning, 2017). The niche theory posits that deterministic processes such as competition, facilitation, predation, resource differentiation, and other environmental filters determine community assembly by causing niche differentiation (Dini-Andreote et al., 2015). The neutral theory hypothesizes that stochastic processes such as colonization, dispersal, priority effects, and ecological drift regulate the assembly and functioning of ecological communities (Dini-Andreote et al., 2015). Disease-induced changes in plant performance lead to cascading variations in the rhizosphere environment that greatly influence the deterministic-stochastic balance of the rhizosphere microbiome (Wen et al., 2022; Zhang et al., 2022). Although this is obviously known, it has been rarely tested and examined in detail. It can be hypothesized that pathogen invasion, as a selection pressure acting upon the rhizosphere microbiome by changing plant performance, would deterministically drive the compositional variation of the rhizosphere microbiome. Pathogen invasion is commonly seen alongside changes in rhizosphere microbial diversity (Wei et al., 2018; Yuan et al., 2018; Shi et al., 2019). It can therefore be concluded that pathogen invasion would affect microbial interactions that depend on the types and abundances of microorganisms present (Weiland-Brauer, 2021). In addition, the dynamic universality (whether interactions among microbes and with their environment are consistent across hosts or whether each individual's microbiota follows its own rules) of the plant root's bacterial microbiome is also yet to be assessed (Bashan et al., 2016). These knowledge gaps limited the effective management of soil and plant health through strategies exploiting the plant-associated microbiome. Furthermore, while the community functions of natural microbial communities are always redundant (Louca et al., 2018; Li P. et al., 2019), pathogen invasion would change the functions in rhizosphere microbes, as community function and assembly are tightly coupled (Xun et al., 2019; Luan et al., 2020).

**Figure 1:**
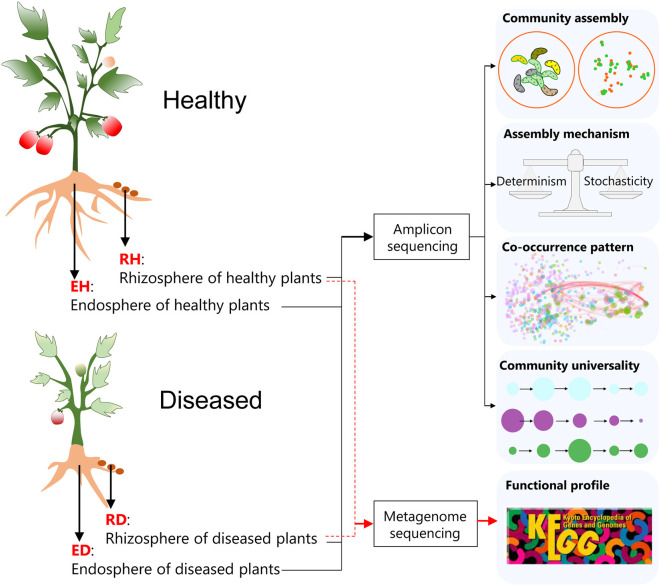
A flow diagram of the key experimental settings in the current study. Healthy and diseased (bacterial wilt disease, BWD) tomato (*Solanum Lycopersicum* L.) plants were harvested, and the variations in the rhizosphere and root endosphere microbial communities were subsequently investigated using amplicon sequencing and shotgun metagenome sequencing. RH, rhizosphere of healthy plants; RD, rhizosphere of diseased plants; EH, endosphere of healthy plants; ED, endosphere of diseased plants.

*Ralstonia solanacearum* is one of the most economically important phytopathogens since the lethal BWD it causes can lead to devastating impacts on many plant species. Tomato (*Solanum lycopersicum* L.) is one of the most widely grown vegetable crops (Fibiani et al., 2022). BWD in tomatoes caused by *R. solanacearum* leads to high annual production losses. A deep understanding of how the plant-associated microbiome responds to BWD may help in the development of environmental-friendly BWD control strategies. In this study, the plant-associated microbiome was investigated using sequencing to determine the differences in the communities of healthy and diseased tomato plants. Plants were harvested and their rhizosphere and root endosphere microbial communities were investigated, examining how plant-associated microbiome assembly, interactions in the plant-associated microbiome, and the functional potentials of plant-associated microbes are affected by pathogen invasion (Figure 1).

## Materials and methods

### Collection of soil and plant samples

Sampling was performed on 15 September 2021 at plastic greenhouses with tomatoes growing for six contiguous seasons, located in Hengxi Town, Nanjing city, Jiangsu province, China (118°46'E, 31°43'N). The sample site has a typical subtropical monsoon climate with a mean annual precipitation of 1,106 mm and a mean annual temperature of 15.5°C. The typical soil is udic argosol. The cultivars (“Hezuo 908”) were infected by the pathogen *R. solanacearum* naturally and randomly. Twelve tomato plants that showed bacterial wilt symptoms (75–100% of leaves wilted or dead) were collected from four plastic greenhouses, and 12 non-infected plants were harvested as controls (Figure 1). Three healthy and three diseased plants were taken from each greenhouse at the setting stage, ensuring the chosen healthy plants were not spatially close to the diseased ones. Rhizosphere soil samples from healthy (RH) and diseased plants (RD) were gently collected; after uprooting the plants, excess soil was first gently shaken from the roots, and then the remaining soil attached to the roots was considered rhizosphere soil (Kibbey and Strevett, 2019). Root tissues of healthy (EH) and diseased plants (ED) were collected to investigate the microbial communities in the root endosphere (Figure 1).

### DNA extraction

Total genomic DNA was extracted from both the rhizosphere soil (0.5 g fresh soil from each sample) and the root tissue (1 g fresh plant tissue from each sample) samples using the FastDNA SPIN Kit for Soil (MP Biomedicals, Solon, OH, USA) following the manufacturer's instructions, except for modifications to homogenization when extracting DNA from the endosphere. Before extracting, the surface of the root tissues was sterilized. First, soil on the root surface was removed by rinsing it with water. After this, the tissues were cut into 0.5 cm sections and submerged in 70% ethanol for 5 min, 6% sodium hypochlorite for 3 min, 70% ethanol for 30 s and then washed with sterile H_2_O five times (Ruiz-Perez et al., 2016). Liquid nitrogen grinding was done in a sterile mortar and pestle for root tissue homogenization.

DNA quality and concentration were measured using gel electrophoresis (0.8% agarose gel) and the Qubit dsDNA HS Assay Kit (Thermo Scientific, Rockford, IL, USA), respectively, on a Qubit 3.0 fluorometer.

### Amplicon sequencing and data processing

The primers 515F (5′-GTGCCAGCMGCCGCGG-3′) and 907R (5′- CCGTCAATTCMTTTRAGTTT-3′) targeting the V4–V5 region of the 16S rRNA gene were used to analyze the rhizosphere bacterial community. The primers 799F (5′-AACMGGATTAGATACCCKG-3′) and 1193R (5′-ACGTCATCCCCACCTTCC-3′) targeting the bacterial V5–V7 regions of the 16S rRNA gene were used for endosphere bacterial community analysis. For the polymerase chain reaction (PCR), a 20-μL reaction mixture containing 10 μL of 2 × SYBR Premix Ex TaqTM (Takara, Dalian, China), 0.5 μL of each primer (10 μM), and 10 ng of template DNA was cycled as follows: 3 min at 95°C; 27 cycles of 30 s at 95°C, 30 s at 55°C, and 45 s at 72°C; a final extension of 10 min at 72°C. PCR reactions were performed in triplicate. The PCR product was extracted from 2% agarose gel, purified using the AxyPrep DNA Gel Extraction Kit (Axygen Biosciences, Union City, CA, USA) according to manufacturer's instructions, and quantified using a Quantus™ Fluorometer (Promega, USA). The purified amplicons were then sequenced by Majorbio Bio-Pharm Technology Co., Ltd. (Shanghai, China) on Illumina MiSeq PE300.

After sequencing, the raw 16S rRNA gene sequencing reads were demultiplexed, quality-filtered by fastp version 0.20.0 (Chen et al., 2018), and merged using FLASH version 1.2.7 (Magoc and Salzberg, 2011) as described in a previous study (Chu et al., 2020). The resulting data were then processed in the QIIME2 (Quantitative Insight into Microbial Ecology toolkit, version 2021.8) pipeline (Bolyen et al., 2019). DADA2 (Callahan et al., 2016) was deployed to remove noise and obtain absolute sequence variants (ASVs) with *denoise-single* plugin. Considering that the amplicons were obtained from the rhizosphere and endosphere with different primers, operational taxonomic units (OTUs) were chosen from ASVs at 97% sequence identity against the SILVA 138 SSURef NR 99 full-length sequences database (Quast et al., 2013) using the vsearch *cluster-features-closed-referenc*e plugin. OTUs assigned to chloroplasts, mitochondria, or archaea were removed, and the corresponding representative sequences were also filtered. After quality filtering, a total of 1,688,642 high-quality 16S rRNA reads were generated, which were finally clustered into 3,331 OTUs.

### Shotgun metagenome sequencing and data processing

To evaluate the impact of BWD on the community function of tomato bacterial microbiomes, rhizosphere DNA samples were mixed for shotgun metagenome sequencing. The construction of metagenome libraries was performed using the NEB Next^®^ Ultra™ DNA Library Prep Kit for Illumina^®^ (New England Biolabs, MA, USA). Eight DNA samples were sequenced as 150 bp paired-end reads on the Illumina NovaSeq 6000 platform at Houze Biological Technology Co. (Hangzhou, China) with an average of 20 Gb raw data per sample. Trimming and quality filtering were performed using Trimmomatic 0.39 (Bolger et al., 2014). To avoid any disturbance, reads mapped to the host reference genome (*Solanum lycopersicum*, RefSeq ID GCF_000188115.5) were removed by Bowtie 2 (version 2.4.1; Langmead and Salzberg, 2012). The remaining reads were *de novo* assembled into contigs using metaSPAdes v.3.13.0 (parameters: K-mer Sizes = 21, 33, 55; Nurk et al., 2017). Generated contigs longer than 800 bp were selected to obtain the coding sequences (CDSs) and corresponding amino acid (AA) sequences using the predicting function of Prodigal (version 2.6.1, Hyatt et al., 2010). All predicted genes were then clustered to a non-redundant gene catalog by using CD-HIT version 4.8.1 (Li and Godzik, 2006) with the identity cutoff at 0.95. The quantification of 12,594,607 non-redundant genes in each sample was performed using Salmon (Patro et al., 2017). Functional annotation was carried out with DIAMOND (Buchfink et al., 2015) against the Kyoto Encyclopedia of Genes and Genomes (KEGG, version 94.2) database (Kanehisa et al., 2012), resulting in 8,040 KEGG orthology (KO) functional categories and 501 KEGG pathways.

### Statistical analysis

To determine the diversity in the four groups of samples (RH, RD, EH, and ED), the alpha (Shannon and Chao1 index) and beta (Bray–Curtis dissimilarities) diversity indices were calculated with the “vegan” package in R (Dixon, 2003) using the rarefied microbial abundance table. The differences in alpha diversity were tested using the Wilcoxon rank-sum tests using the base R package “stats” (version 4.1.1) wilcox.test function. Non-metric multidimensional scaling (NMDS) and non-parametric multivariate analysis of variance (ADONIS; Anderson, 2001) were used to examine the microbial community dissimilarity between the four groups. To analyze taxonomic differences in rhizosphere and endosphere bacterial abundance, a linear discriminant analysis (LDA) effect size (LEfSe) analysis was performed on the amplicon dataset at the genus level (Galaxy web application, http://huttenhower.sph.harvard.edu/galaxy/; Segata et al., 2011). NMDS was also applied to determine whether the microbiomes were functionally distinct between different groups.

The modified normalized stochasticity ratio (MST) based on Bray–Curtis dissimilarity was used to identify the bacterial community assembly processes, with 50% as the threshold for determining the dominance of deterministic (MST < 50%) or stochastic processes (MST > 50%; Ning et al., 2019). A neutral community model (NCM, Sloan et al., 2006) was also used to cross-check the results of the MST analysis. To assess the universality of microbial dynamics, a dissimilarity–overlap curve (DOC) analysis was conducted using the R package “DOC” with a bootstrap value of 200 (Bashan et al., 2016). The overlap was defined as the fraction of shared taxa between two communities in the same group. Dissimilarity refers to differences in shared taxa relative abundance. The dissimilarity and overlap of all sample pairs were plotted to generate a dissimilarity–overlap curve (DOC) with a non-parametric regression and bootstrap sampling procedure. Universal dynamics exist only when a negative correlation is detected between dissimilarity and overlap and the DOC inflection point occurs where the slope is negative. The fraction of points after the inflection point is termed *Fns*.

To infer the co-occurrence and mutual exclusion patterns, the CoNet plugin in Cytoscape version 3.7.1 (Shannon et al., 2003) was used to calculate multiple correlations and similarities between the microbial OTUs. To decrease the number of false positives, all taxa below a minimum occurrence of six were combined across the samples into a garbage taxon, and the Benjamini–Hochberg procedure was adopted to adjust the *P*-values. The co-occurrence between taxa was considered valid when the *P*-value (adjusted) was below 0.05 (Benjamini and Hochberg, 1995) and the correlation threshold was above 0.7. Network images were generated with Gephi (version 0.9.2; Heymann, 2009) with the Fruchterman-Reingold layout. Topological parameters of networks were also calculated with Gephi.

Functional diversity, including alpha and beta diversity, was also calculated using the “vegan” package in R. Principal coordinate analysis (PCoA) combined with ADONIS was applied to examine functional gene dissimilarity between RH and RD using “vegan” with the Bray–Curtis dissimilarity metric. Differential analysis of functional genes and KEGG pathways was carried out using a generalized linear model (GLM) in R package “edgeR” (Robinson et al., 2010) and STAMP (Parks et al., 2014) with Welch's *t-test*, and all *P*-values were corrected for a false discovery rate (*FDR*) of 0.01 using the Benjamini–Hochberg algorithm.

## Results

### Diversity and community composition

**Figure 2:**
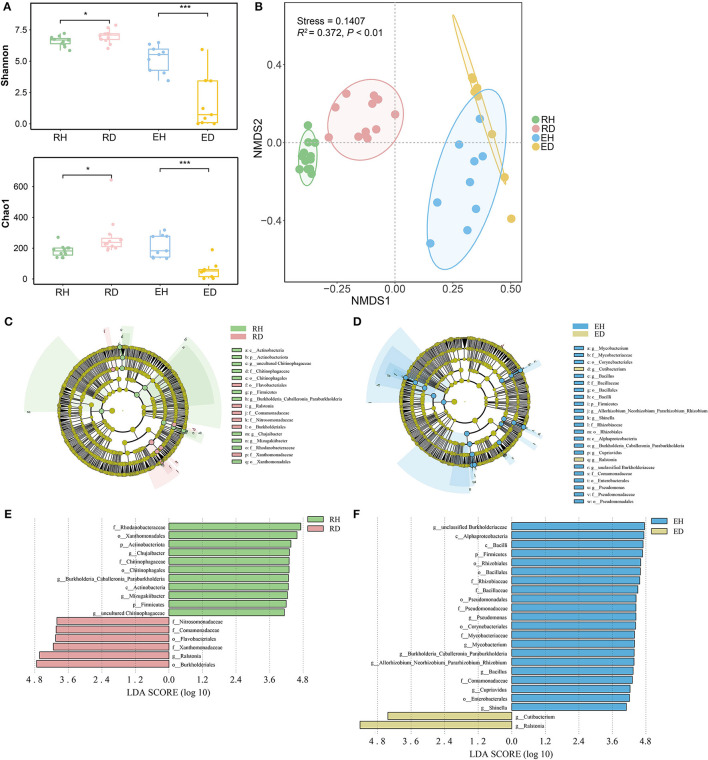
Microbiome assembly in plant rhizosphere and endosphere. **(A)** Alpha diversity of microbial communities in rhizosphere and endosphere. Significant differences between different groups were obtained using pairwise Wilcoxon tests with *P*-values. *P < 0.05, ***P < 0.001. **(B)** Non-metric multidimensional scaling (NMDS) ordinations of Bray–Curtis dissimilarity matrices with permutational analysis of variance (PERMANOVA, *R*^2^= 0.372, *P* < 0.001). The potential biomarkers were defined by LEfSe. Cladogram for taxonomic representation of significant differences between **(C)** RH and RD, and **(D)** EH and ED. The colored nodes from the inner to the outer circles represent taxa from the phylum to the genus level. The significantly different taxa are signified by different colors representing the four groups. Histogram of the LDA scores for differentially abundant features between **(E)** RH and RD, and **(F)** EH and ED. The threshold on the logarithmic LDA score for discriminative features was set to 4.0. RH, rhizosphere of healthy plants; RD, rhizosphere of diseased plants; EH, endosphere of healthy plants; ED, endosphere of diseased plants.

At the phylum level, *Proteobacteria, Bacteroidota*, and *Actinobacteriota* were dominant across all rhizosphere and endosphere samples (Supplementary Figure 1). Bacterial alpha diversity in RH (Shannon index/Chao1 index: 6.70/183.0) was significantly lower than that in RD (Shannon index/ Chao1 index: 7.08/237.5, *P* < 0.001), while bacterial diversity in EH (Shannon index/Chao1 index: 5.52/184.5) was significantly higher than that in ED (Shannon index/Chao1 index: 0.71/52.0, *P* < 0.01, Figure 2A). Non-metric NMDS analysis based on Bray–Curtis dissimilarity showed that the samples clustered well and separated according to the different sample groups (Figure 2B). The ordination plot indicated that microbial community composition was distributed according to the groups (Figure 2B). ADONIS further demonstrated that there was a significant difference in community composition between different groups (*R*^2^ = 0.372, *P* < 0.001). ANOSIM analysis indicated that the compositional variation (indicated by Bray–Curtis dissimilarity) was lower in both RH and EH compared to RD (*R* = 0.469, *P* = 0.001) and RH (*R* = 0.424, *P* = 0.002; Supplementary Figure 2).

LEfSe analysis was conducted to explore the indicator taxa in the four groups. In the rhizosphere, the phyla *Actinobacteriota* and *Firmicutes*, orders *Xanthomonadales* and *Chitinophagales*, and genera *Chujaibacter, Burkholderia, Caballeronia, Paraburkholderia, Mizugakiibacter*, and uncultured *Chitinophagaceae* were enriched in RH. Orders *Burkholderiales* and *Flavobacteriales*, family *Xanthomonadaceae*, and genus *Ralstonia* were greatly enriched in RD compared to RH (Figures 2C, E). In EH, the most significantly different taxa were phylum *Firmicutes*, classes *Alphaproteobacteria* and *Bacilli*, orders *Rhizobiales, Bacillales, Pseudomonadales, Corynebacteriales*, and *Enterobacterales*, families *Rhizobiaceae, Bacillaceae, Pseudomonadaceae, Mycobacteriaceae*, and *Comamonadaceae*, and genera *unclassified Burkholderiaceae, Pseudomonas, Mycobacterium, Burkholderia Caballeronia Paraburkholderia, Allorhizobium, Neorhizobium, Pararhizobium, Rhizobium, Bacillus, Cupriavidus*, and *Shinella*. Compared to EH, genera *Ralstonia* and *Cutibacterium* were significantly enriched in ED (Figures 2D, F).

### Community assembly mechanisms

**Figure 3:**
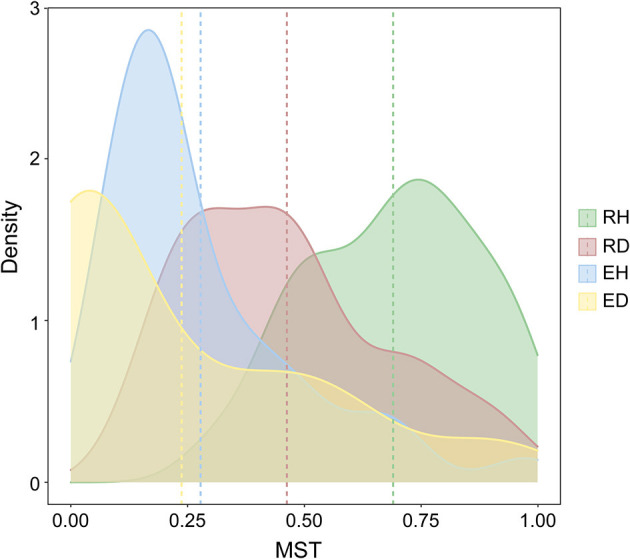
Modified normalized stochasticity (MST) ratios estimated for bacterial community assembly mechanism. The four groups are represented by different colors. The vertical dashed lines represent the mean MST values of different groups. RH, rhizosphere of healthy plants; RD, rhizosphere of diseased plants; EH, endosphere of healthy plants; ED, endosphere of diseased plants.

The modified normalized stochasticity ratio (MST) index was calculated to evaluate the importance of stochasticity and determinism for bacterial community assembly. The average MST values in RH, RD, EH, and ED were 0.690, 0.462, 0.278, and 0.238, respectively (Figure 3). In addition, we further assessed the contribution of the stochastic process to community assembly by the neutral community model (NCM). The NCM model performance was indicated by *R*^2^, where a higher *R*^2^ (close to 1) indicates a better neutral fitting (more stochastically assembled). RH had a higher NCM model *R*^2^ in comparison with RD (*R*^2^ = 0.607 vs. *R*^2^ = 0.475, Supplementary Figure 3).

### Microbial co-occurrence patterns

**Figure 4:**
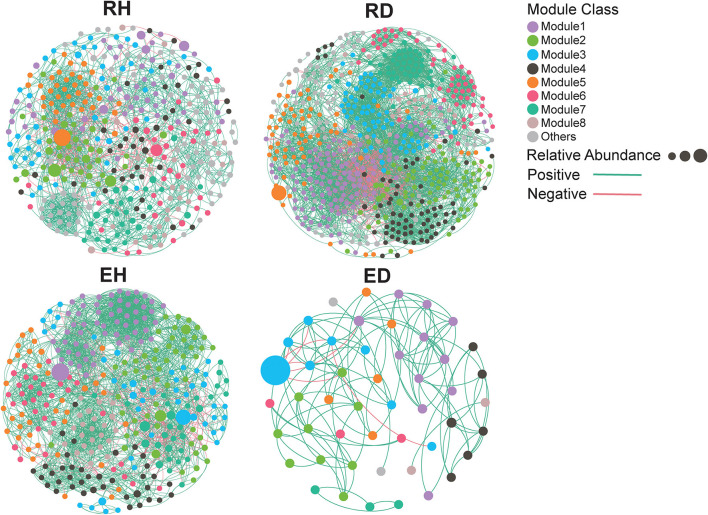
Co-occurrence network for bacterial communities. Each node represents an OTU, and the relationship between two OTUs in the network was shown by an edge (Coefficient *R* ≥ 0.7, significant *P* ≤ 0.01). The nodes are colored according to module classes. Node size reflects the relative abundance of an OTU. The edge color represents positive (green) or negative (red) correlation. RH, rhizosphere of healthy plants; RD, rhizosphere of diseased plants; EH, endosphere of healthy plants; ED, endosphere of diseased plants.

To determine the effect of BWD on microbial co-occurrence patterns, networks based on correlation relationships were constructed (Figure 4). Based on complexity-denoting network topological parameters, higher node (RH/RD: 381/621) and edge (RH/RD: 3654/11958) numbers and smaller betweenness centrality (RH/RD: 0.0017/0.0012) were identified in the RD network compared to the RH network (Table 1). In contrast, higher node (EH/ED: 322/49) and edge (EH/ED: 5478/260) numbers and smaller betweenness centrality (EH/ED: 0.0026/0.0525) were recorded in the EH network compared to that recorded in the ED network (Table 1). The topological parameters, clustering coefficient (RH/RD: 0.466/0.567), average degree (RH/RD: 9.591/19.256), and average path length (RH/RD: 4.11/2.68), were also assessed, and a higher clustering coefficient and average degree but lower average path length were observed in the RD network compared to that in the RH network (Table 1).

**Table 1:** Topology properties of the RH, RD, EH, and ED networks.

**Parameter**	**RH**	**RD**	**EH**	**ED**
No. of nodes	381	621	322	49
No. of edges	3,654	11,958	5,478	260
Linkage density	4.8	9.63	8.51	2.65
No. of positive edges/proportion (%)	3,458 (86%)	10,678 (89%)	5,126 (94%)	246 (95%)
No. of negative edges/proportion (%)	496 (14%)	1,280 (11%)	352 (6%)	14 (5%)
Clustering coefficient	0.466	0.567	0.63	0.8
Avg. degree	9.591	19.256	17.012	5.306
Betweeness centrality	0.0017	0.0012	0.0026	0.0525
Closeness centrality	0.2587	0.2829	0.3001	0.5765
Modularity	0.673	0.65	0.67	0.624
Network density	0.025	0.031	0.053	0.111
Average path length	4.11	3.68	3.38	2.61

RH, rhizosphere of healthy plants; RD, rhizosphere of diseased plants; EH, endosphere of healthy plants; ED, endosphere of diseased plants.

### Effects of BWD on community universality

Universal dynamics denotes when communities in the same group share the same interaction rules. The rules are supported only if the shared taxa have the same relative abundances (Bashan et al., 2016). To assess whether there are universal dynamics in samples that belonged to the same group, we performed a dissimilarity–overlap curve (DOC) analysis. Here, the overlap indicates the fraction of the shared taxa between two communities in the same group. Dissimilarity refers to differences in the composition of the shared taxa based on relative abundance. The dissimilarity and overlap of all sample pairs are then plotted to generate a DOC with a non-parametric regression and a bootstrap sampling procedure. Universal dynamics were determined only when a negative correlation was detected between dissimilarity and overlap, and the inflection point occurred in DOC where the slope was negative. The fraction of points after the inflection point is termed as *Fns*.

**Figure 5:**
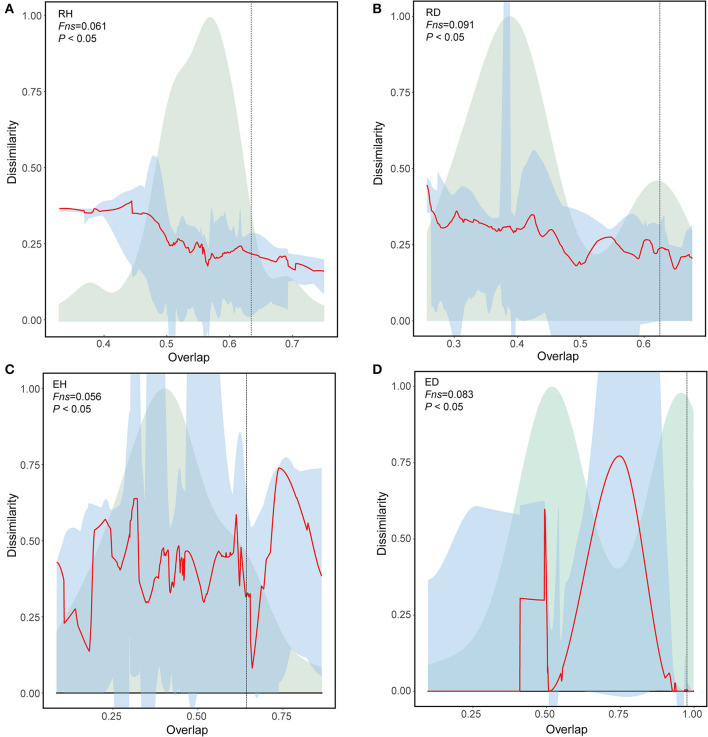
Universal ecological dynamics of RH **(A)**, RD **(B)**, EH **(C)**, and ED **(D)** microbiome. The ecological universality of microbiome was assessed using dissimilarity-overlap curves (DOC). DOCs are in red, the distribution density of sample pair overlap is in light green, and the point at which the DOC first becomes negative is marked by a vertical dashed line (chosen by median of 200 bootstraps). A higher *Fns* value indicates higher ecological universality (host-independent) of the metacommunity. RH, Rhizosphere of healthy plants; RD, Rhizosphere of diseased plants; EH, Endosphere of healthy plants; ED, Endosphere of diseased plants.

The DOCs had significant negative slopes, with an *Fns* of 6.1% for RH community comparisons (Figures 5A, B); this was lower than that of the RD community comparisons (9.1%). Similarly, the DOCs for EH comparisons had significant negative slopes with an *Fns* of 5.6%, which was also lower than those observed for the diseased ED community comparisons (8.3%; Figures 5C, D).

### Determining the functional profile of the rhizosphere microbiome

**Figure 6:**
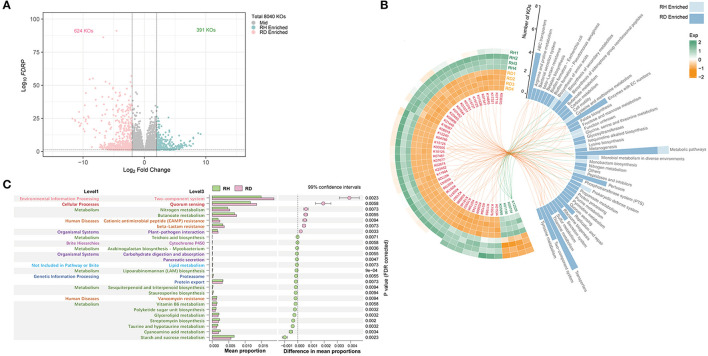
Functional profile of rhizosphere microbiome. Significantly (*P* < 0.01) different A, B KO functional categories and C pathways between RH and RD. All KO functional categories are depicted in **(A)**, and the differential KO functional categories were evaluated using the generalized linear model (GLM) edgeR approach. The abundant differential KO functional categories (relative abundance > 0.08%) are described in the heatmap **(B)**, and the involved pathways are counted in the histogram and are linked by lines. KO functional categories that were significantly enriched in RH or RD were separately analyzed for KEGG pathway enrichment; those pathways that did not belong to microorganisms were removed; all significantly enriched pathways are described in **(C)**. The pathway difference between RH and RD was quantified using a two-tailed Wilcoxon test, and the corrected *P*-values were shown. RH, rhizosphere of healthy plants; RD, rhizosphere of diseased plants.

The metagenomic functional profiling yielded a total of 8,040 KO functional categories. PCoA and Adonis reflected significant differences in microbiome functional profiles between RH and RD (*R*^2^ = 0.71, *P* < 0.05, Supplementary Figure 4D). A total of 1,015 significantly different KOs were detected, of which 391 were found to be significantly enriched in RH and 624 were enriched in RD (Figure 6A).

To clarify which significant KOs were predominant, KOs with relative abundance >0.08% were used for statistical analysis. This resulted in 48 KOs, four of which were significantly enriched in RH; the remaining 44 were significantly enriched in RD. K01156 (*res*, type III restriction enzyme [EC:3.1.21.5], prokaryotic defense system, mean 0.055%) was most abundant in RH, followed by K12526 (*lysAC*, bifunctional diaminopimelate decarboxylase/aspartate kinase, mean 0.026%), K05998 (pseudomonalisin [EC:3.4.21.100], peptidases, and inhibitors, mean 0.022%), and K09704 (mean 0.018%; Figure 6B). In RD, the top three KOs were K07481 (transposase, IS5 family, mean 0.287%), K15125 (*fhaB*, filamentous hemagglutinin, glycosaminoglycan binding proteins, mean 0.235%), and K11904 (*vgrG*, type VI secretion system secreted protein VgrG, mean 0.166%). When the pathways involved with these significantly differential KOs were mapped, KOs enriched in RH were mainly assigned to KEGG pathways “Prokaryotic defense system,” “Peptidases and inhibitors,” “Monobactam biosynthesis,” “Glycine, serine, and threonine metabolism,” and “cysteine and methionine metabolism.” Except for pathway “Metabolic pathways,” which was shared with RH, KOs enriched in RD mainly mapped into pathways “Transporters,” “ABC transporters,” “Enzymes with EC numbers,” “Two-component system,” “Secretion system,” “Replication and repair,” and “biosynthesis of siderophore group non-ribosomal peptide” (Figure 6B).

A total of 19 enriched KEGG pathways were detected in RH and seven were enriched in RD. Despite the pathways enriched in RH exhibiting a diversified pattern, pathways with high relative abundance were principally those involved in metabolism and human diseases (vancomycin resistance). In RD, the pathway “Two-component system” involved in environmental information processing was most abundant, followed by “Quorum sensing” involved in cellular processes. Pathways “Nitrogen metabolism” and “Butanoate metabolism” were more abundant in RD than in RH. Furthermore, pathways “Cationic antimicrobial peptide (CAMP) resistance”/“beta-Lactam resistance” involved in human diseases and “Butanoate metabolism” and “Plant–pathogen interaction” involved in organismal systems also showed significantly higher relative abundance in the RD samples (Figure 6C).

## Discussion

Using a field sampling approach, the present study revealed multifaceted disease-induced variations in the community assembly and functions of plant-associated microbiomes, which may provide new insights into the role of pathogen invasion in altering the functioning of plant-associated microbiomes in soil ecosystems.

### BWD increased diversity of the rhizosphere microbiome

The disease occurs commonly alongside changes in microbial diversity in the rhizosphere (Wei et al., 2018; Yuan et al., 2018; Shi et al., 2019). Contrasting previous reports showing pathogen invasion led to a decline in microbial diversity, the results of this study showed that the alpha diversity of diseased plants (RD) was significantly higher than that of healthy ones (RH; Figure 2A). This demonstrated that pathogen invasion may not necessarily suppress microbial diversity in the rhizosphere (Gibbons et al., 2016); on the contrary, the diverse nutrients released by the damaged roots may have the potential to feed more microbial species in the rhizosphere, leading to a diversity increase after pathogen invasion. Similarly, beta diversity also increased with pathogen infection (Figure 2B), indicating that pathogen invasion disrupted host control of the rhizosphere microbiome (Wei et al., 2018; Wen et al., 2020). This is inconsistent with the prediction of the Anna Karenina principle, which suggested that the microbiome of diseased hosts may display greater compositional or functional variation compared to healthy ones (Arnault et al., 2023). Environmental perturbations such as pathogen invasion could lead to the development of new niches, and microbes nearby could then colonize the rhizosphere opportunistically, resulting in higher beta diversity (Macke et al., 2017; Yu et al., 2020; Lin et al., 2022).

### BWD promoted microbial co-occurrence complexity and altered the relative contribution of deterministic processes

Using the ecological null model, the relative contribution of deterministic processes in influencing bacterial community assembly increased under diseased conditions (Figure 3 and Supplementary Figure 3B). The deterministic process is mainly derived from environmental filtering and species competition that influence the occurrence and abundance of species (Zhou and Ning, 2017). The enhanced deterministic processes observed after pathogen invasion may be jointly attributed to the drastic environmental changes as well as to different biological interactions that then occurred (Zhang et al., 2022). Roots damaged by pathogen invasion can release rich nutrients into the rhizosphere; consequently, many microbial species that favor copiotrophic conditions would be subsequently enriched, while some oligotrophic species would be selectively excluded. For example, the genus *Ralstonia* is mainly composited of copiotrophic species that prefer nutrient-rich conditions and was found to be significantly enriched in diseased plants (Li et al., 2021). The “cry for help” hypothesis suggests that many plant-beneficial microbial species, especially those with the ability to antagonize pathogens, would be selectively recruited by plants after pathogen invasion, contributing to the deterministic processes in the diseased plant rhizosphere (Gao et al., 2021; Arnault et al., 2023).

While co-occurrence does not directly reflect interaction, it allows the construction of a linkage between community assembly processes and co-occurrence patterns. Using the co-occurrence network analysis, pathogen invasion was found to increase the network complexity of the plant-associated microbiome (Table 1), implying that pathogen invasion potentially promoted microbial interactions. In addition to network analysis, the universal dynamics of the plant-associated microbiome were analyzed using DOCs, which can also reflect microbial interactions (Bashan et al., 2016). A higher *Fns* value was found for diseased plants than for healthy plant-associated microbiomes, suggesting that microbes in diseased plants interacted more closely. These results consistently indicated that pathogen invasion promoted microbial interactions in the rhizosphere and endosphere (Figure 5), which may contribute to the deterministic assembly. It should be noted that the tightened microbial interactions may also be linked to plant disease. A community with tight and complex interactions always has lower community stability, since resonance is more likely to occur (Coyte et al., 2015; de Vries et al., 2018). As a consequence, it can be inferred that pathogens may have better chances to colonize and proliferate in these less stable environments. Therefore, future experiments testing plant disease incidence and community stability in complex microbial communities will be necessary to discern their relative importance in soil and plant health.

### BWD induced changes in microbiome functions

Using shotgun metagenome sequencing, we investigated how pathogen invasion affects the functioning profile of the rhizosphere microbiome. Metagenomic analysis indicated that several genes essential for the pathogenicity of *R. solanacearum* involved in pathways “Two-component system” and “Secretion system” (Genin and Denny, 2012), such as *fhaB and vgrG*, were enriched in RD (Figure 6B). A higher relative abundance of K05998 (pseudomonalisin [EC:3.4.21.100], peptidases, and inhibitors) was found in RH. Peptidases have been reported to be potential biocontrol factors participating in the predation of *Myxococcus xanthus* on *R. solanacearum* (Dong et al., 2022; Figure 6B). An important differential pathway worth noting is “Quorum sensing,” which was found to be significantly enriched in the diseased rhizosphere (Figure 6C). Quorum sensing can coordinate the expression of specific genes in multiple pathogens and regulate pathogenic performance (Bassler, 1999). It can therefore be speculated that other microorganisms may induce pathogenic *Ralstonia* to cause plant disease, although this needs to be demonstrated by further experimentation (Shi et al., 2019; Li M. et al., 2022). In contrast, some plant-beneficial pathways such as “streptomycin biosynthesis” were found to be significantly enriched in the healthy plant rhizosphere (Figure 6C). Streptomycin is an aminoglycoside antibiotic that inhibits protein synthesis and targets the 30S ribosomal protein RpsL. Streptomycin has been used to control multiple plant bacterial diseases and can antagonize multiple phytopathogens such as *Pseudomonas aeruginosa* and *R. solanacearum* (Lee et al., 2018; Attia et al., 2022).

## Conclusion

This study aimed to reveal how plant-associated microbiomes respond to plant disease. The results demonstrated that pathogen invasion enhanced the bacterial deterministic processes, promoted microbial co-occurrence complexity and ecological universality, and modified the functional profile of the plant-associated microbiome. This study broadens the understanding of the relationships between plant disease and the plant microbiome and provides novel insights into BWD occurrence.

## Data availability statement

The data presented in the study are deposited in the NCBI's archival repository, accession number PRJNA917505.

## Author contributions

PL and JJ designed the work. LK and TL conducted the experiments. LK analyzed the data and wrote the manuscript. BW participated in revising the manuscript. JP and JL took part in sample collection. All authors contributed to the study and approved the final submitted version.
