# Supplementary material for: Diseased-induced multifaceted variations in community assembly and functions of plant-associated microbiomes
Source: Front Microbiol. 2023 Mar 16;14:1141585. doi: 10.3389/fmicb.2023.1141585 (PMC10060855; doi:10.3389/fmicb.2023.1141585)
Supplement: Supplementary file 1 [file Data_Sheet_1.docx]

**Supplementary Materails for**

**Diseased-induced** **multifaceted variations in community assembly and function of plant-associated microbiome**

Lu Kuang^1^, Ting Li^1^, Baozhan Wang^1^, Junwei Peng^2^, Jiangang Li^2^, Pengfa Li^1*^, Jiandong Jiang^1*^

**Affiliations:**

^1^ Key Lab of Microbiology for Agricultural Environment, Ministry of Agriculture, College of Life Sciences, Nanjing Agricultural University, Nanjing 210095, China.

^2^ State Key Laboratory of Soil and Sustainable Agriculture, Institute of Soil Science, Chinese Academy of Sciences, Nanjing 210008, China

*** Correspondence:**

Pengfa Li: pfli@njau.edu.cn

Jiandong Jiang: [jiang_jjd@njau.edu.cn](mailto:jiang_jjd@njau.edu.cn)

College of Life Sciences, Nanjing Agricultural University, WeiGang Road 1, Nanjing 210095, China

**
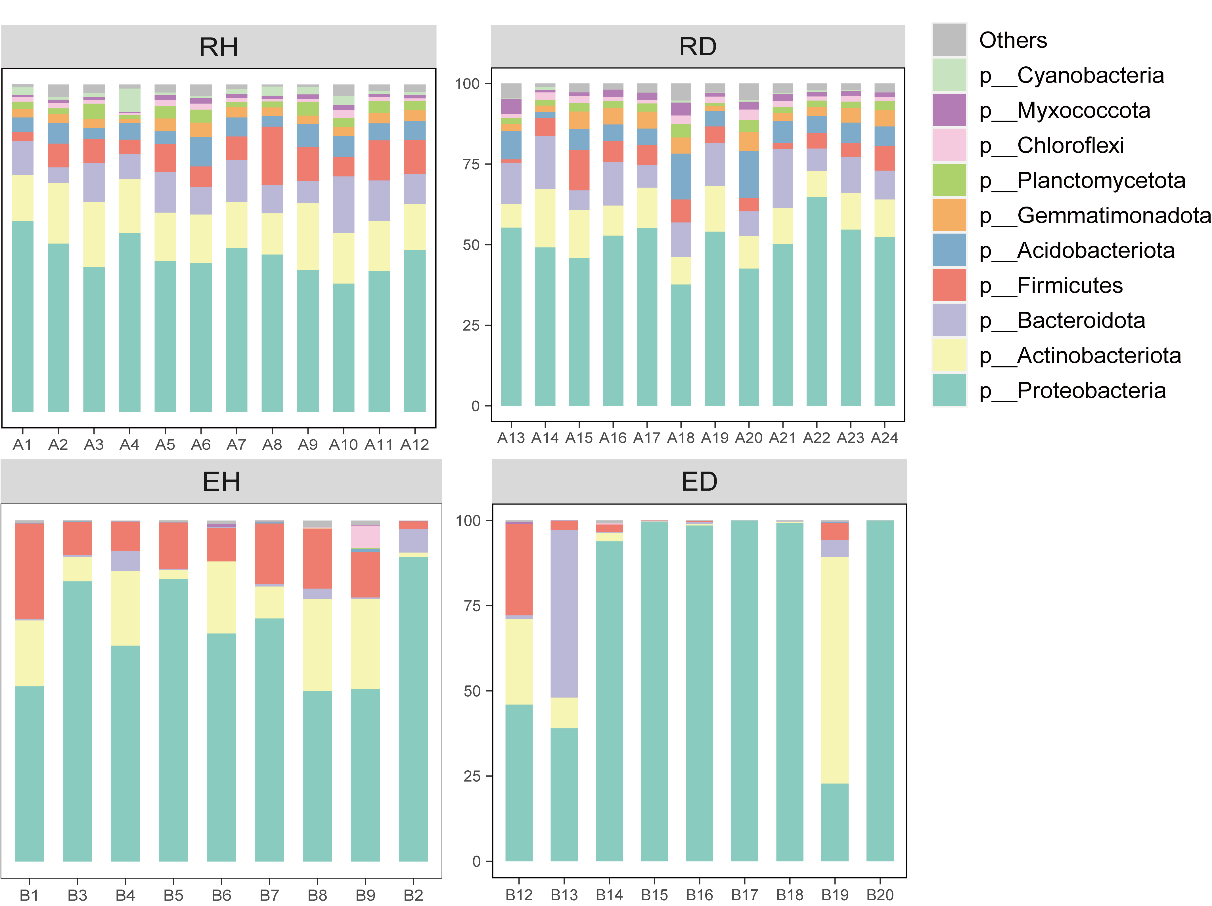
**

**Figure S1** Community structure and composition of RH, RD, EH and ED. Relative abundances of the top 10 phyla by composition in RH, RD, EH and ED. RH: Rhizosphere of healthy plants; RD: Rhizosphere of diseased plants; EH: Endosphere of healthy plants; ED: Endosphere of diseased plants.


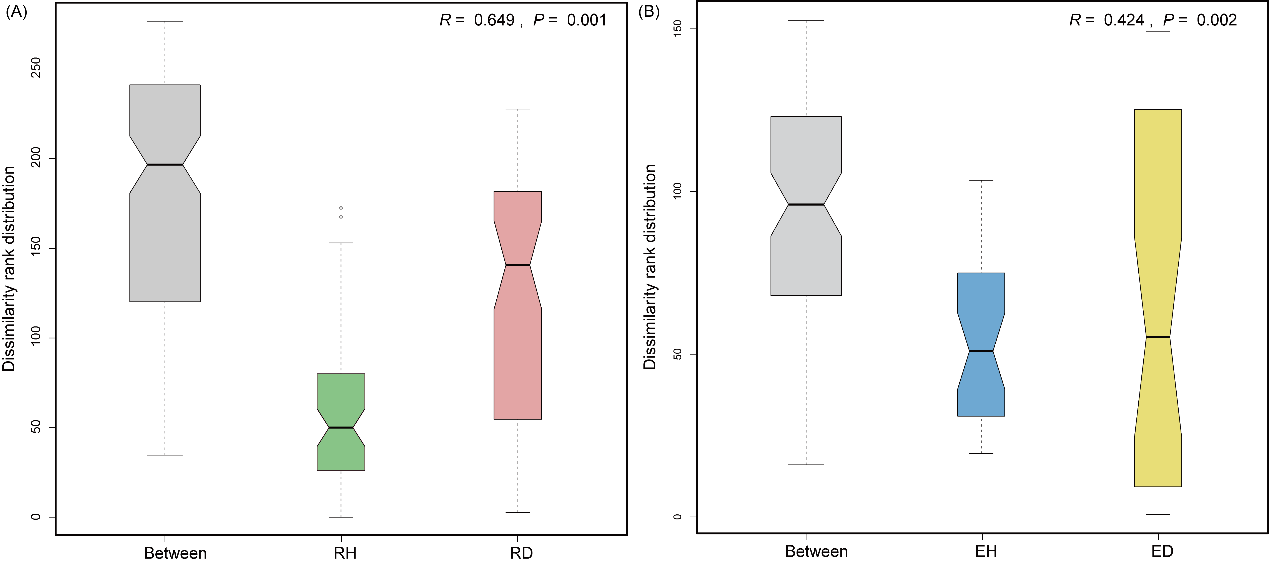


**Figure S2** Anosim analysis results. Between represents the difference between groups; others are within groups; the greater the distance is, the greater the difference is; and the thickness is the sample size. RH: Rhizosphere of healthy plants; RD: Rhizosphere of diseased plants; EH: Endosphere of healthy plants; ED: Endosphere of diseased plants.


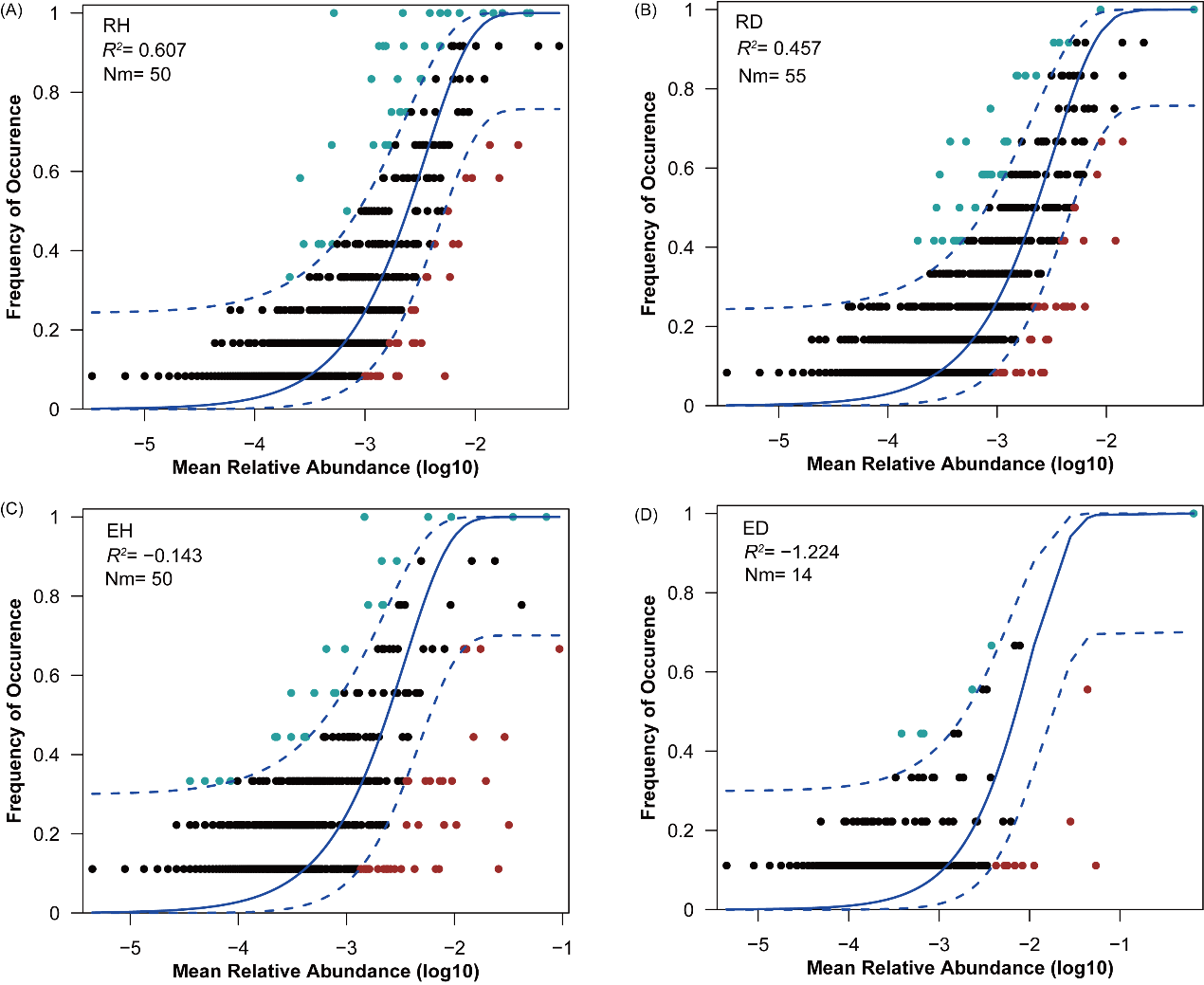


**Figure S3** Fit of the neutral community model (NCM) of community assembly. The predicted occurrence frequencies for RH (A), RD (B), EH (C), and ED (D) communities, respectively. The solid blue lines indicate the best fit to the NCM as in Sloan et al., and the dashed blue lines represent 95% confidence intervals around the model prediction. OTUs that occur more or less frequently than predicted by the NCM are shown in different colors. Nm indicates the metacommunity size times immigration, R^2^ indicates the fit to this model. RH: Rhizosphere of healthy plants; RD: Rhizosphere of diseased plants; EH: Endosphere of healthy plants; ED: Endosphere of diseased plants.


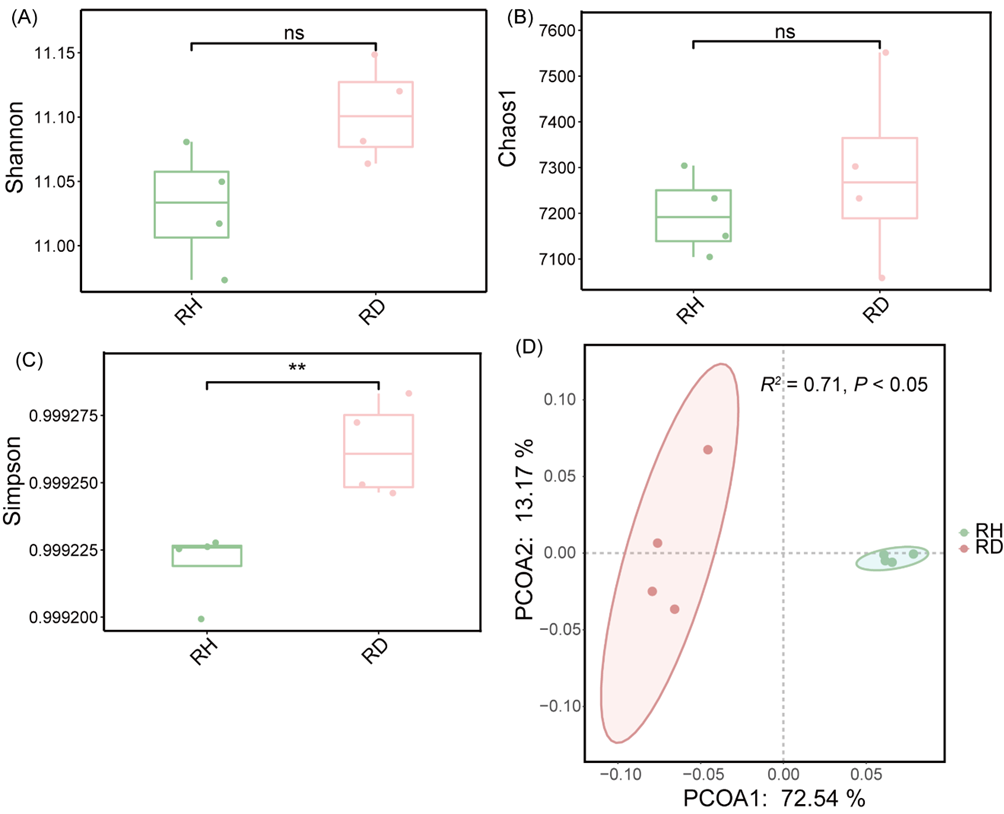


**Figure S4** Microbiome functional diversity. Number of alpha diversity indices (A) Shannon index, (B) Chao1 index, (C) Simpson index using relative abundance of KO functional categories. Significant differences within RH and RD were obtained using pairwise Wilcoxon tests with *P*-values. ns represents no significance, ** represents *P* < 0.01. (C) Principal coordinates analysis (PCoA) of Bray–Cutis dissimilarity matrices with permutational analysis of variance (PERMANOVA, *R^2^*= 0.71, *P* < 0.005). RH: Rhizosphere of healthy plants; RD: Rhizosphere of diseased plants.
